# Cloning, purification, kinetic and anion inhibition studies of a recombinant β-carbonic anhydrase from the Atlantic salmon parasite platyhelminth *Gyrodactylus salaris*

**DOI:** 10.1080/14756366.2022.2080818

**Published:** 2022-05-30

**Authors:** Ashok Aspatwar, Harlan Barker, Heidi Aisala, Ksenia Zueva, Marianne Kuuslahti, Martti Tolvanen, Craig R. Primmer, Jaakko Lumme, Alessandro Bonardi, Amit Tripathi, Seppo Parkkila, Claudiu T. Supuran

**Affiliations:** a Faculty of Medicine and Health Technology, Tampere University, Tampere, Finland; b Ecology and Genetics, University of Oulu, Oulu, Finland; c Department of Biology, University of Turku, Turku, Finland; d Department of Computing, University of Turku, Turku, Finland; e Organismal and Evolutionary Biology Research Programme, University of Helsinki, Helsinki, Finland; f Institute of Biotechnology, Helsinki Institute of Life Science, University of Helsinki, Helsinki, Finland; g Department of Neuroscience, Psychology, Drug Research and Child’s Health, Section of Pharmaceutical and Nutraceutical Sciences, University of Florence, Sesto Fiorentino, Italy; h Department of Zoology, University of Lucknow, Lucknow, India; i Fimlab Ltd, Tampere University Hospital, Tampere, Finland

**Keywords:** Carbonic anhydrase, *Gyrodactylus salaris*, kinetics, anion inhibitors, sulphamic acid

## Abstract

A β-class carbonic anhydrase (CA, EC 4.2.1.1) was cloned from the genome of the Monogenean platyhelminth *Gyrodactylus salaris,* a parasite of Atlantic salmon. The new enzyme, GsaCAβ has a significant catalytic activity for the physiological reaction, CO_2_ + H_2_O ⇋ HCO_3_^−^ + H^+^ with a k_cat_ of 1.1 × 10^5^ s^−1^ and a k_cat_/K_m_ of 7.58 × 10^6^ M^−1^ × s^−1^. This activity was inhibited by acetazolamide (K_I_ of 0.46 µM), a sulphonamide in clinical use, as well as by selected inorganic anions and small molecules. Most tested anions inhibited GsaCAβ at millimolar concentrations, but sulfamide (K_I_ of 81 µM), *N,N*-diethyldithiocarbamate (K_I_ of 67 µM) and sulphamic acid (K_I_ of 6.2 µM) showed a rather efficient inhibitory action. There are currently very few non-toxic agents effective in combating this parasite. GsaCAβ is subsequently proposed as a new drug target for which effective inhibitors can be designed.

## 1. Introduction

**Figure 1.**
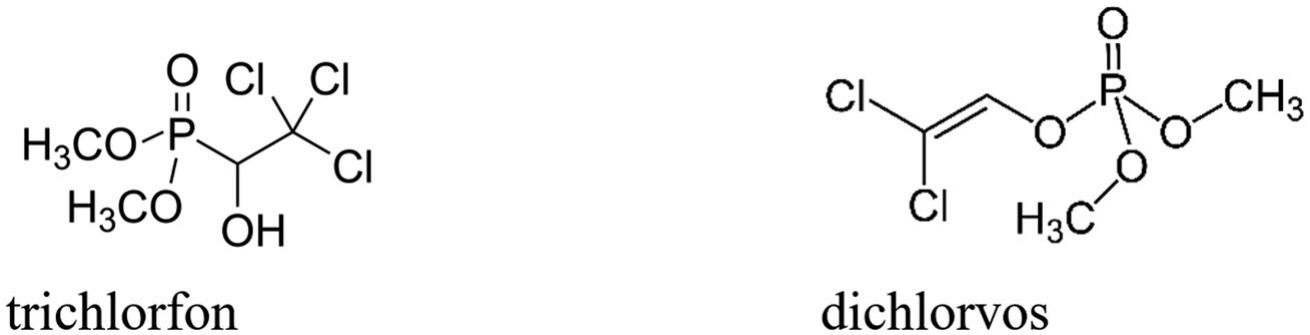
The acetylcholinesterase inhibitors trichlorfon and dichlorvos.

*Gyrodactylus salaris* is a flatworm (platyhelminth) parasite belonging to the Monogeneans group, which are hermaphrodite ectoparasites found on the gills, fins, or skin of fish^1^^,^^2^. They do not need an intermediate host for infecting a range of fish species, some of which possess significant commercial status, such as the Atlantic salmon (*Salmo salar*) and related species^3^^,^^4^. The presence of this parasitic pathogen has been reported in 19 countries across Europe and has already produced catastrophic losses of Atlantic salmon mainly in Norway starting in the 1970s and in Russian Karelia since 1992^1–4^. This small (0.5 mm) parasite attacks the host by attaching its anterior end to the fish through secretions from the cephalic glands, and then releasing a digestive solution rich in proteolytic enzymes which dissolves the fish skin, inducing the formation of large wounds which favour secondary infections^5^. A variety of inorganic and organic compounds, among which are salt (NaCl), hypochlorite, permanganate, aluminium salts, praziquantel, levamisole, mebendazole, toltrazuril, etc., have been tested for efficacy against a broad spectrum of monogenean species, including *G. salaris*, but only trichlorfon and dichlorvos (Figure 1) showed some efficacy^6^^,^^7^. However, both compounds act as irreversible organophosphoric acetylcholinesterase inhibitors, showing thus a rather high toxicity for all vertebrates, not only for fish^8^.

Novel potential drug targets present in the proteome of this parasite have been recently explored. This includes excretory/secretory proteins involved in host invasion and colonisation^9^, with a flatworm host invasion and colonisation, such as a flatworm protease^9^. However, no pharmacological inhibitors were reported so far.

Considering the fact that the metalloenzyme carbonic anhydrase (CA, EC 4.2.1.1) has a fundamental role in many organisms, as it catalyses the hydration of CO_2_ to bicarbonate and protons^10–17^, and also that such enzymes were already investigated in parasitic (*Ascaris lumbricoides*, *Schistosoma* spp., etc.)^18–24^ and non-parasitic (*Caenorhabditis elegans*) worms^25–27^ for their inhibition with various classes of inhibitors, it appeared of interest to investigate whether this enzyme is also present in *G. salaris*. Here we report the cloning, characterisation and anion inhibition studies of a β-class CA (GsaCAβ) encoded in the genome of *G. salaris*, which we propose as a potential drug target for the management of this parasitic fish disease.

## 2. Materials and methods

### 2.1. Construction of vector for recombinant protein production

**Figure 2.**
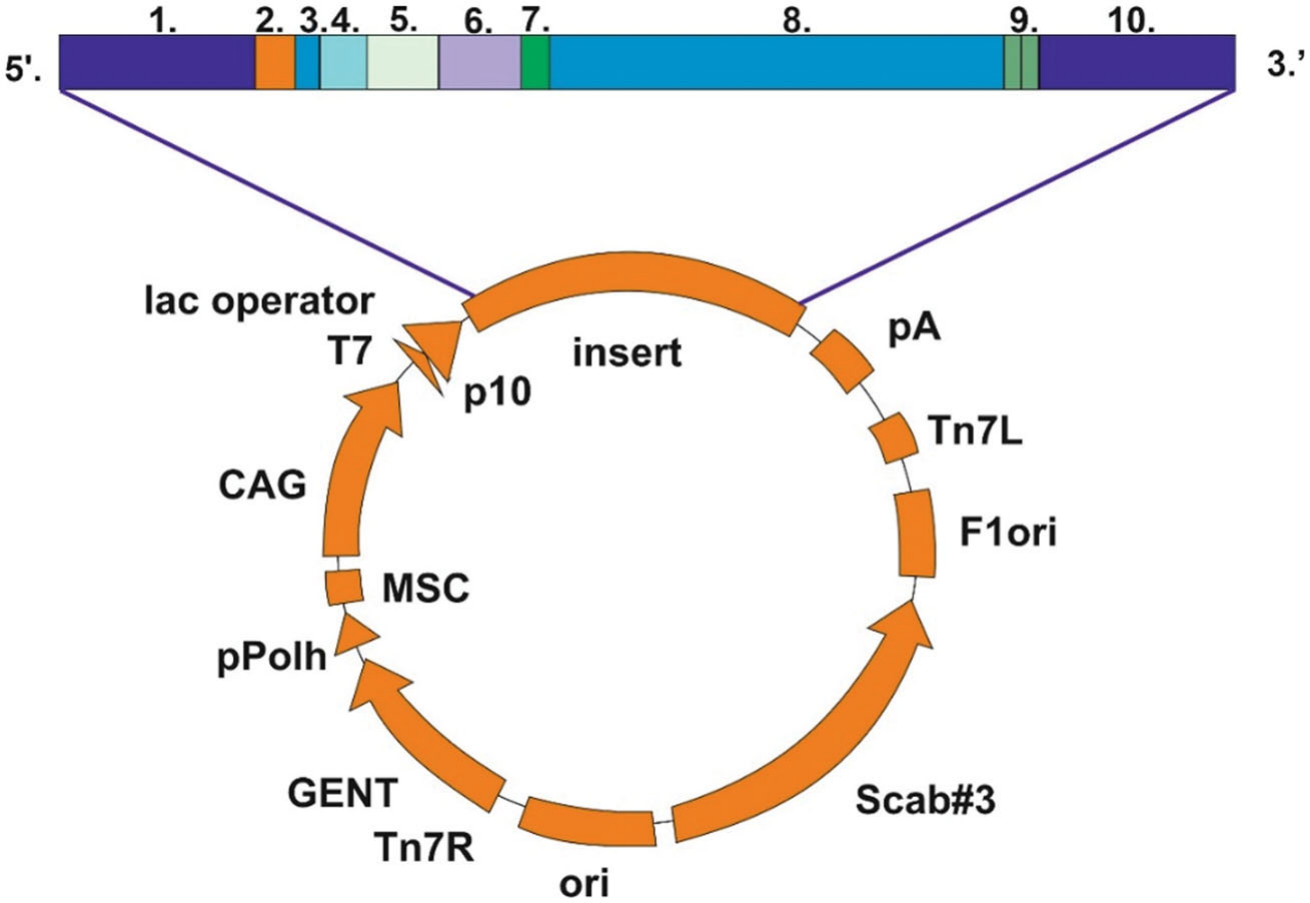
Schematic presentation of pBVboostFG expression vector designed for production of recombinant protein. The insert contains: 1. attL1, 2. Shine-Dalgarno, 3. Kozak, 4. Met-Ser-Tyr-Tyr, 5. 6 x His, 6. Asp-Tyr-Asp-Ile-Pro-Thr-Thr, 7. Lys-Val, 8. β-CA gene of *G. salaris* gene of interest, 9. Two stop codons, and 10. AttL2.

A nearly complete GsaCAβ sequence was obtained from transcriptome data produced at the University of Turku. The open reading frame produced a translation of 229 amino acids, 165 of which were supported by genome data from the University of Oulu. A BLAST search at NCBI^28^ suggested that the sequence is close to full length, and therefore the existing sequence was only extended by an ATG codon at the beginning of the transcript and a stop codons (TAA TAG) at the end. This sequence of the β-CA was inserted in a pBVboost vector construct for the production of recombinant protein (Figure 2). The construct was obtained by GeneArt (Invitrogen, Regensburg, Germany). The sequence of the β-CA was modified accordingly to produce the protein in bacterial (*Escherichia coli*) cells.

### 2.2. Transformation of plasmid vector into BL21 cells

The construct of the β-CA sequence from the freeze-dried plasmid supplied by GeneArt was prepared according to the instructions of the manufacturer. The BL21 Star™ (DE3) cells were stored at −80 °C cells (Invitrogen, Carlsbad, CA, USA) and thawed by keeping them on ice. After thawing the competent cells, 25 µl of the cell suspension and 1 µl of the reconstituted plasmid, were transferred into a 1.5 ml centrifuge tube. The suspension was incubated on ice for 30 min. Heat shock was performed by keeping the tube in 42 °C water for 30 s, and transferred immediately on ice for 2 min. 125 µl of SOC Medium (Invitrogen, Carlsbad, CA, USA) was added to the microcentrifuge tube containing the transformed cells, and the tube was incubated at 37 °C for 1 h with shaking (200 rpm). The agar plates containing gentamycin were stored at 37 °C before the transformation. 20 µl or 50 µl of cell suspension described above were spread onto each plate, and the plates were incubated overnight at 37 °C . A volume of 5 ml preculture was prepared by inoculating single colonies from growth plates onto an LB medium with gentamycin (ratio 1:1000), being then incubated overnight at 37 °C with constant shaking of 200 rpm.

### 2.3. Production of GsaCAβ recombinant protein

Protein production was carried out according to pO-stat fed batch protocol as described earlier with some modifications^29^. The fermentation medium contained no glycerol, as the cell line used did not require it. For induction of the culture 1 mM IPTG was used after 12 h of starting the fermentation. The temperature was decreased to 25 °C at the time of the induction. Cell growth was stopped after 12 h of the induction with the OD 34 (A600). The cells were collected by centrifugation and the weight of the cell pellet was recorded. The fermentation was carried out at the Protein Services core facility of Tampere University (https://www.tuni.fi/en/research/protein-services). The cell pellet was suspended in 150 ml of binding buffer containing 50 mM Na_2_HPO_4_, 0.5 M NaCl, 50 mM imidazole, and 10% glycerol (pH 8.0). Cell pellet was lysed with sonication (5 min, 30 s off, 20 s on) into the lysing buffer and centrifuged at 20 000×*g*/15 min. The suspension was homogenised using EmulsiFlex-C3 (AVESTIN, Ottawa, Canada) homogeniser. The lysate was centrifuged at 13 000×*g* for 15 min at 4 °C, and the clear supernatant was mixed with HisPur™ Ni-NTA Resin (Thermo Fisher Scientific, Waltham, MA, USA) and bound to the resin for 2 h at room temperature on the magnetic stirrer. The obtained resin was washed with the binding buffer and collected onto an empty column with an EMD Millipore™ vacuum filtering flask (Merck, Kenilworth, NJ, USA) and filter paper. The protein from the resin was eluted using 50 mM Na_2_HPO_4_, 0.5 M NaCl, 350 mM imidazole, and 10% glycerol (pH 7.0). The protein was re-purified with TALON^®^ Superflow™ cobalt resin (GE Healthcare, Chicago, IL, USA). The eluted protein fractions were diluted with binding buffer (50 mM Na_2_HPO_4_, 0.5 M NaCl, and 10% glycerol pH 8.0), so that the imidazole concentration was under 10 mM. The protein binding and elution were performed as described above. The purity of the protein was determined with gel electrophoresis (SDS-PAGE) and visualised with PageBlue Protein staining solution (Thermo Fisher Scientific, Waltham, MA, USA). Protein fractions were pooled and concentrated according to the protocol (https://store.repligen.com/products/floatalyzer) (8–10 kD). Buffer exchange in 50 mM TRIS (pH 7.5) was done using the same centrifugal concentrators. The His-tag was cleaved from the purified protein by Thrombin CleanCleave Kit (Sigma-Aldrich, Saint Louis, MO, USA), according to manufacturer’s manual.

### 2.4. CA activity and inhibition measurements

An Applied Photophysics stopped-flow instrument has been used for assaying the CA catalysed CO_2_ hydration activity^30^. Phenol red at a concentration of 0.2 mM was used as pH indicator, working at the absorbance maximum of 557 nm, with 10 mM TRIS (pH 8.3) as buffer, and in the presence of 10 mM NaClO_4_ for maintaining constant the ionic strength, following the initial rates of the CA-catalysed CO_2_ hydration reaction for a period of 10–100 s. The CO_2_ concentrations ranged from 1.7 to 17 mM for the determination of the kinetic parameters and inhibition constants. For each inhibitor, at least six traces of the initial 5–10% of the reaction have been used for determining the initial velocity. The uncatalyzed rates were determined in the same manner and subtracted from the total observed rates. Stock solutions of inhibitors (10–20 mM) were prepared in distilled-deionized water and dilutions up to 0.01 µM were done thereafter with the assay buffer. Inhibitor and enzyme solutions were preincubated together for 15 min at room temperature prior to assay, in order to allow for the formation of the enzyme-inhibitor complex. The inhibition constants^31–37^ represent the mean from at least three different determinations. GsaCAβ concentration in the assay system was 14.3 nM.

### 2.5. Reagents

Anions and small molecules were commercially sold reagents of the highest available purity from Sigma-Aldrich (Milan, Italy). The purity of tested compounds was higher than 99%.

### 2.6. Phylogenetic analysis

A BLAST search was performed on the UniPROT webserver (https://www.uniprot.org/blast/) with the novel GsaCAβ sequence as a query and all settings as default. The top 250 closely related sequences, and their annotations (species, phyla), were taken for further analysis.

The 250 β-CAs were clustered to 80% similarity with the "cluster fast" algorithm of the USEARCH tool (version 11.0.667)^38^ and 111 sequences representing the centroids of clusters resulted. To this list the novel *G. salaris* β-CA was added, and a custom Python script was then used to further filter out sequences that did not contain both canonical β-CA amino acid motifs (CxDxR and HxxC), resulting in a total of 104 β-CA sequences which were then aligned with Muscle (version 5.1) using all default settings^39^.

The alignment was reduced to a total of 62 conserved amino acid residues which were identified using GBlocks (version 0.91 b)^40^ with parameters “-t = p -b2 = 6 -b3 = 20 -b4 = 2 -b5 = h -d = y -v = 240”. Model testing was performed to identify the best evolutionary model for analysis of the target sequences using ModelFinder^41^, with the best model determined to be “LG + I+G4”. A maximum likelihood phylogenetic analysis was performed using the IQTree software (version 2.0.3)^42^, with parameters set to "-alrt 100000 -bb 100000 -nt AUTO -m LG + I+G4" and all other options run as default. A consensus tree was generated from the 100 000 bootstrap replicates, with a final log-likelihood value of −4717.575. The tree was then visualised using a custom Python script utilising the ETE Toolkit Python library^43^.

The code and workflow used to perform these analyses are provided at https://github.com/thirtysix/Aspatwar.Gsalaris_BCA (Supplemental data).

### 2.7. Subcellular localizations

TMHMM 2.0^44^ (https://services.healthtech.dtu.dk/service.php?TMHMM-2.0) was used for the prediction of transmembrane helices. TargetP 2.0^45^ (https://services.healthtech.dtu.dk/service.php?TargetP-2.0) was used for the prediction of various N-terminal targeting peptides, with parameters for non-plant proteins. Finally, we performed predictions for multiple subcellular localizations with DeepLoc 1.0^46^ (https://services.healthtech.dtu.dk/service.php?DeepLoc-1.0).

### 2.8. Multiple sequence alignment

Our protein sequence of GsaCAβ was used as a query in BLAST searches^28^ at NCBI (https://blast.ncbi.nlm.nih.gov/Blast.cgi). Searches were made limited to metazoa, except vertebrates, in the nr database with wordsize 3 and scoring matrix BLOSUM45, otherwise default parameters. The results were filtered for at least 90% query coverage. Seven homologs were selected based on taxonomical diversity and model organism status from the results with E value cut-off 1e-28 (top 242 hits). Details of all the hits are given in https://bit.ly/3JNRb7i (Supplemental data).

Sequences were aligned with Clustal Omega^47^ at EBI (https://www.ebi.ac.uk/Tools/msa/clustalo/) with number of combined iterations = 3, otherwise default parameters. Espript 3^48^ at https://espript.ibcp.fr/ESPript/cgi-bin/ESPript.cgi was used in visualising the sequence alignment result. Our AlphaFold model for GsaCAβ (see below) was used for the display of secondary structures above the alignment. The threshold for boxing nearly conserved residues was set to 80%.

### 2.9. Molecular modelling

All operations with 3 D protein structure models and molecular visualisation were performed using ChimeraX (daily build 1.4.dev202202030703), developed by the UCSF Resource for Biocomputing, Visualisation, and Informatics (San Francisco, California, USA), supported in part by the National Institutes of Health^49^.

A 3D model of GsaCAβ was created with AlphaFold^50^ using the ChimeraX interface to submit the prediction to run at Google Colab. This model was compared to a crystallographic structure of the pea β-CA, PDB 1EKJ^51^ by 3 D superimposition using the MatchMaker tool of ChimeraX with an iteration cut-off of 1.5 Å for pruning residue pairs in fitting.

## 3. Results and discussion

### 3.1. Sequence analysis of GsaCAβ, comparison with other β-CAs, and recombinant protein production

The partial GsaCAβ transcript sequence discovered in this study, of 687 nucleotides, has been submitted to European Nucleotide Archive (ENA) and assigned the accession number OW406882. The translated peptide of 229 amino acids can be found in the CDS/translation feature of the entry at http://www.ebi.ac.uk/ena/data/view/OW406882.

**Figure 3.**
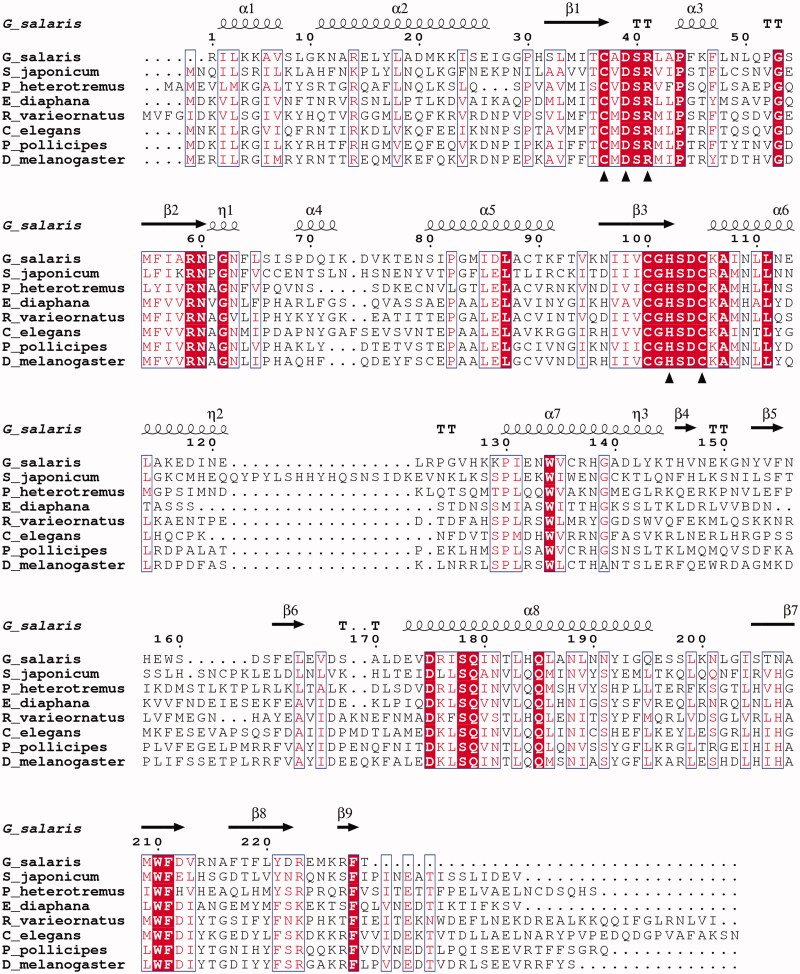
Alignment of GsaCAβ sequence with β-CA sequences of other metazoans. The conserved hallmark catalytic-site sequences of β-CAs, CXDXR and HXXC, are shown with black triangles (C: Cysteine, D: Aspartic acid, H: Histidine, R: Arginine, X: any residue). Columns with fully conserved residues are shown as red with white letters. Boxed columns denote positions in which at least 80% of residues are of similar type. The top line shows secondary structures derived from our GsaCAβ model. α: α-helices; β: β-strands; η: 3_10_-helices; T: turns.

The bioinformatic analyses of *G. salaris* genomic data revealed the presence of a novel CA sequence that resembled the β-CAs from other metazoan species, as seen in the small MSA of Figure 3. The sequences in the MSA are detailed in Table 1. The GsaCAβ sequence is moderately similar to the β-CAs of other metazoans (Figure 3 and Table 2), with regions of highest similarity at 32 to 115 and at 176 to 192 (GsaCAβ numbering). The hallmark motifs CxDxR and HxxC of the active site of β-CAs, in which the cysteines and histidine coordinate the catalytic zinc ion, are conserved in GsaCAβ (shown by triangles in Figure 3). It is notable that the sequence identity is nearly constant between GsaCAβ and β-CAs from widely different groups of animals, as seen in Tables 1 and 2. Sequence identity was 29.8% ± 1.4% (mean ± SD) in the whole set of 431 metazoan Blast hits. The MSA of Figure 3 contains 26 fully conserved positions and 59 partially conserved (boxed) positions along the GsaCAβ sequence of 229 residues, in total 85, or 37.1% of the positions.

**Table 1.** Sequences in the multiple sequence alignment of Figure 3.

NCBI accession	Scientific name	Group
KAH8855123.1	*Schistosoma japonicum*	Platyhelminthes
KAF5400048.1	*Paragonimus heterotremus*	Platyhelminthes
XP_020895242.1	*Exaiptasia diaphana*	Sea anemones
GAU97340.1	*Ramazzottius varieornatus*	Tardigrades
NP_741809.1	*Caenorhabditis elegans*	Nematodes
XP_037075907.1	*Pollicipes pollicipes*	Crustaceans
NP_649849.1	*Drosophila melanogaster*	Insects

**Table 2.** Percent identity matrix of the aligned protein sequences of Figure 3, as computed by Clustal Omega

		1	2	3	4	5	6	7	8
1	*G. salaris*	100							
2	*S. japonicum*	30	100						
3	*P. heterotremus*	30	38	100					
4	*E. diaphana*	29	28	29	100				
5	*R. varieornatus*	32	30	29	36	100			
6	*C. elegans*	30	31	33	41	39	100		
7	*P. pollicipes*	31	34	31	44	47	46	100	
8	*D. melanogaster*	28	30	30	41	43	50	60	100

Analysis of the GsaCAβ sequence with TMHMM 2.0 suggests that no transmembrane helices are present. TargetP 2.0 predicts a cytoplasmic (“other”) localisation, from among the choices of secreted, mitochondrial, or other. DeepLoc 1.0, on the other hand, predicts mitochondrial localisation from among 10 different localizations with a likelihood of 0.77. DeepLoc does not depend solely on N-terminal sequences in its inference, contrary to TargetP 2.0. Furthermore, the authors of TargetP 2.0 state that for non-plant proteins, the most common confusion is between mitochondrial targeting peptides and no targeting peptides^45^. The same article also notes that the second residue in the sequence has a markedly strong predictive value for metazoan mitochondrial targeting peptides. Considering all this, and the fact that our protein sequence is incomplete at the N-terminus, we prefer to accept the prediction of DeepLoc and suggest tentatively a mitochondrial localisation for GsaCAβ. This would also be consistent with mitochondrial localisation observed or predicted for many other metazoan β-carbonic anhydrases (BCAs)^20^^,^^52^^,^^53^.

The sequence comparisons carried out here confirm that GsaCAβ belongs to β-CA enzyme family and could be a potential target for developing suitable inhibitors for control of this parasite in fish culture farms.

**Figure 4.**
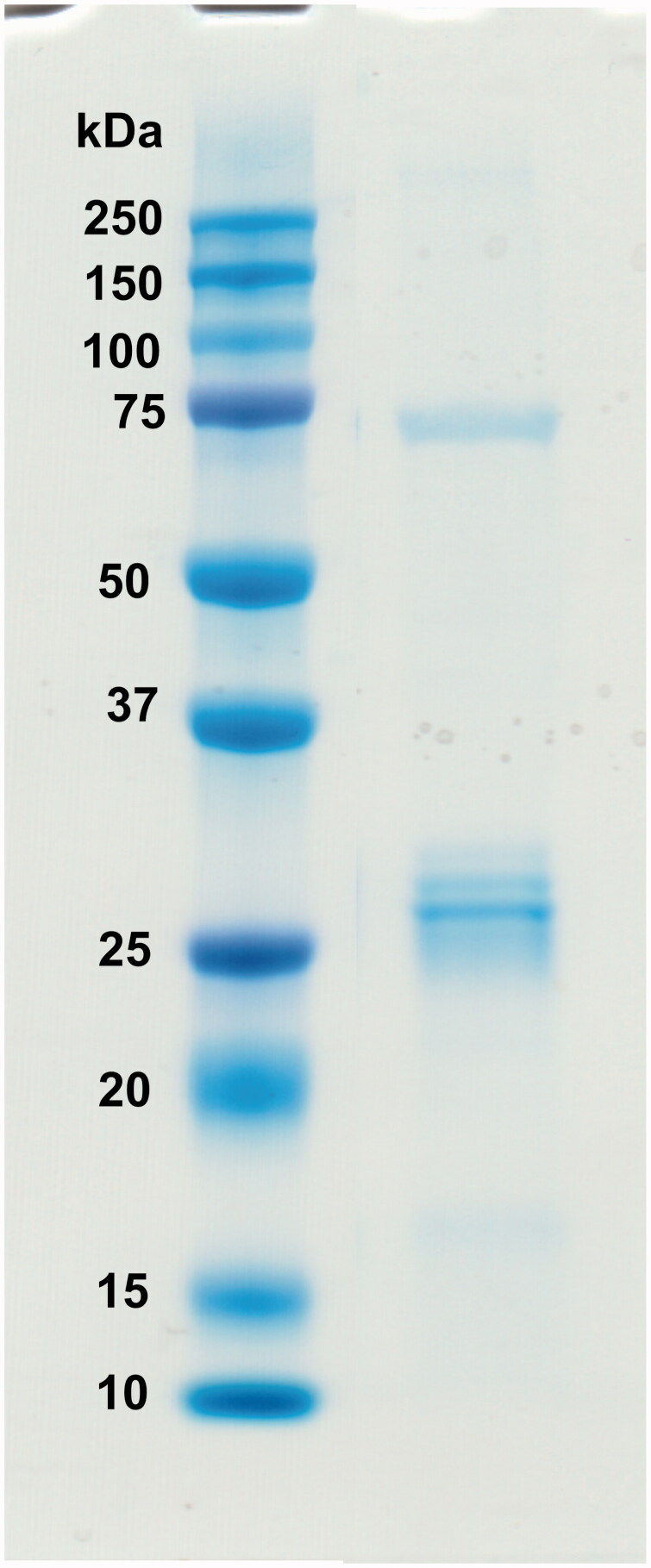
Recombinant GsaCAβ protein analysed on SDS-PAGE. The image shows protein ladder standards (left) and the purified recombinant *G. salaris* β-CA protein (right) showing a molecular mass calculated from mobility of 26.0 kDa.

The recombinant GsaCAβ protein was produced in *E. coli* cells. The purified recombinant protein showed a single band close to the expected size on SDS-PAGE (Figure 4, measured MW 26.0 kDa).

### 3.2. Phylogeny of 131 β-CA sequences

**Figure 5.**
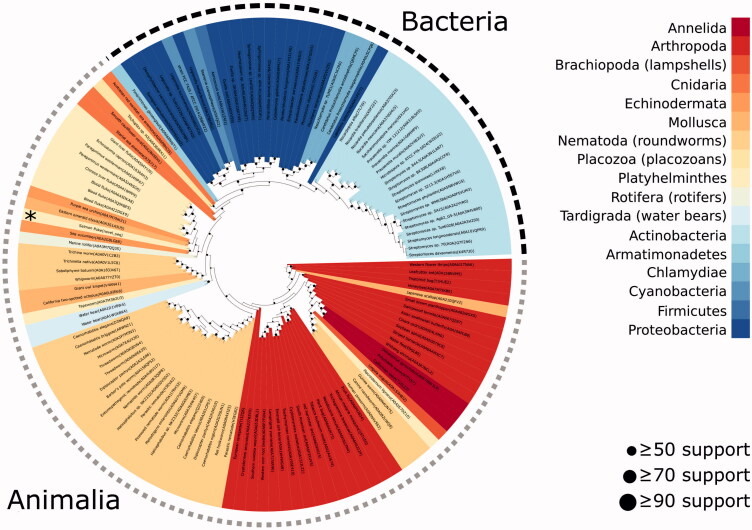
Phylogenetic analysis of β-CAs related to the novel enzyme identified in *G. salaris*.

Phylogenetic analysis was performed using the GsaCAβ sequence together with 130 related β-CA sequences identified via BLAST search. Among all sequences included there were 17 distinct phyla represented, 5 of which came from bacteria (46 sequences) and 12 from animalia (85 sequences). Within animalia, the largest two groups were nematoda (29 sequences) and arthropoda (28 sequences). The most closely related sequence to the novel GsaCAβ was from sea cucumber (A0A2G8LGE8), and together these occurred within a larger clade of 13 total sequences, which included 8 other platyhelminthes. Figure 5 shows the final phylogenetic tree.

A total of 130 βCA sequences identified by BLAST search of the UniProt database were combined with the GsaCAβ sequence and used to perform maximum likelihood phylogenetic analysis (IQTree version 2.0.3)^42^ to identify potential evolutionary relationships. Colours represent the phyla of the species from which each sequence originates. *G. salaris* is marked with an asterisk. Dot sizes in the internal nodes of the tree indicate bootstrap support level of each node.

### 3.2. Catalytic activity of GsaCAβ and its inhibition with acetazolamide

The catalytic activity of the new β-CA, GsaCAβ, was investigated for the physiological reaction catalysed by CAs, CO_2_ hydration to bicarbonate and protons (Table 3). The experiments were performed at a pH value of 8.3 where the active site of these enzymes is open^54–65^. We compared the activity of GsaCAβ with that of other earlier characterised β-CAs from fungi, yeasts and bacteria (Table 3).

**Table 3.** Kinetic parameters for the CO_2_ hydration reaction^30^ catalysed by α- and β-class CA enzymes: the human cytosolic isozymes hCA I and II (α-class CAs) at 20 °C and pH 7.5 in 10 mM HEPES buffer, Can2 (from *Cryptococcus neoformans*), CalCA (from *Candida albicans*), SceCA (from *Saccharomyces cerevisiae*), and Cab (from the archaeon *Methanobacterium thermoautotrophicum*) measured at 20 °C, pH 8.3 in 20 mM TRIS buffer and 10 mM NaClO_4_.

Isozyme	Activity level (s^−1^)	k_cat_ (M^−1^ x s^−1^)	k_cat_/K_m_ (nM)	K_I_ (acetazolamide)
hCA I^a^	moderate	2.0 × 10^5^	5.0 × 10^7^	250
hCA II^a^	very high	1.4 × 10^6^	1.5 × 10^8^	12
Can2^b^	moderate	3.9 × 10^5^	4.3 × 10^7^	10.5
CalCA^c^	high	8.0 × 10^5^	9.7 × 10^7^	132
SceCA ^d^	high	9.4 × 10^5^	9.8 × 10^7^	82
Cab^e^	low	3.1 × 10^4^	1.82 × 10^6^	12100
EcoCAβ^f^	moderate	5.3 × 10^5^	4.10 × 10^7^	227
GsaCAβ^g^	low	1.1 × 10^5^	7.58 × 10^6^	460

^a^Ref. [10,12,13]; ^b^Ref.[54,55]; ^c^Ref. [56–58]; ^d^Ref. [59,60]; ^e^Ref.[61,62]; ^f^Ref. [63–65]; ^g^This work.

Inhibition data with the clinically used sulphonamide, acetazolamide (5-acetamido-1,3,4-thiadiazole-2-sulphonamide), are also provided.

Data presented in Table 3 show that GsaCAβ has a low but significant catalytic activity for the physiological reaction, with a k_cat_ of 1.1 × 10^5^ s^−1^ and k_cat_/K_m_ of 7.58 × 10^6^ M^−1^ × s^−1^ (K_M_ of 14.5 mM), being thus around 12 times less efficient compared to hCA II (Table 3)^10–13^. However, this catalytic activity is relevant, being comparable to that of similar enzymes from well-known bacterial/fungal pathogens (Table 3)^54–65^ where a relevant physiological/pathological role has been demonstrated by using specific inhibitors of enzymatic activity^54–65^. Indeed, inhibition of CAs has been shown to interfere with the growth and virulence of the pathogens^66–68^. Also, in the case of GsaCAβ, the enzymatic activity was inhibited with a K_I_ of 460 nM by the classical sulphonamide inhibitor acetazolamide (Table 3).

### 3.3. Anion inhibition studies of GsaCAβ

A panel of anions and small molecules known for interacting with CAs^69^ were chosen to be tested as inhibitors of GsaCAβ (Table 3).

As seen from the data in Table 4, where the inhibition of the human α-class isoforms hCA I and II was also included for comparison, many inorganic/organic anions and small molecules, such as sulfamide and sulphamic acid, inhibited GsaCAβ. The action of inhibition is presumably through coordination of the molecule to the metal ion in the active site, as with other CAs previously investigated for their interaction with this type of inhibitor^13–17^^,^^54^^,^^55^^,^^70^^,^^71^. The following should be noted regarding the inhibition data of Table 4:

**Table 4.** Anion inhibition data of the β-CA from *G. salaris* and human isoforms hCA I and hCA II as determined by stopped-flow CO_2_ hydrase assay.

	K_I_ (mM)^a^		
Inhibitor^b^	hCA I	hCA II	GsaCAβ
F^−^	>300	>300	5.5
Cl^−^	6	200	3.3
Br^−^	4	63	8.2
I^−^	0.3	26	>50
CNO^−^	0.0007	0.03	1.9
SCN^−^	0.2	1.6	2.7
CN^−^	0.0005	0.02	0.86
N_3_^−^	0.0012	1.51	>50
NO_2_^−^	8.4	63	9.1
NO_3_^−^	7	35	>50
HCO_3_^−−^	12	85	>50
CO_3_^2−−^	15	73	>50
HSO_3_^−−^	18	89	6.2
SO_4_^−^	63	>200	>50
HS^−^	0.0006	0.04	6.9
NH_2_SO_2_NH_2_	0.31	1.13	0.081
NH_2_SO_3_H	0.021	0.39	0.0062
PhAsO_3_H_2_	31.7	49	>50
PhB(OH)_2_	58.6	23	>50
ClO_4_^−^	>200	>200	>50
SnO_3_^2−^	0.57	0.83	0.77
SeO_4_^2−^	118	112	>50
TeO_4_^2−^	0.66	0.92	4.3
OsO_5_^2−^	0.92	0.95	2.5
P_2_O_7_^2−^	25.8	48	>50
V_2_O_7_^2−^	0.54	0.57	4.9
B_4_O_7_^2−^	0.64	0.95	>50
ReO_4_^−^	0.11	0.75	3.8
RuO_4_^−^	0.101	0.69	7.1
S_2_O_8_^2−^	0.107	0.084	0.91
SeCN^−^	0.085	0.086	3.5
NH(SO_3_)_2_^2−^	0.31	0.76	3.1
FSO_3_^−^	0.79	0.46	0.92
CS_3_^2−^	0.0087	0.0088	5.4
EtNCS_2_^−^	0.00079	0.0031	0.067
PF_6_^−^	>50	>50	>50
CF_3_SO_3_^−^	>50	>50	>50

^a^Mean from 3 different assays, by a stopped flow technique (errors were in the range of ± 5–10% of the reported values); ^b^Sodium salts, except sulfamide and phenylboronic acid.

- Anions and small molecules which were non-inhibitory (up to 50 mM) against GsaCAβ included iodide, azide, nitrate, bicarbonate, carbonate, sulphate, phenylarsonic acid, phenylboronic acid, perchlorate, selenate, pyrophosphate, tetraborate, hexafluorophosphate and triflate. Many of these anions/small molecules poorly interact with metal ions in solution or within the metalloenzymes’ active sites (e.g. perchlorate, hexafluorophosphate and triflate)^69^, whereas others (e.g. azide, iodide, phenylarsonic acid, phenylboronic acid, etc.) inhibit with various efficacy CAs belonging to other classes^13^^,^^69–71^. Azide, for example, is a rather efficient hCA I inhibitor, with a K_I_ of 1.2 µM, and has thoroughly been characterised by diverse techniques, including X-ray crystallography^14–17^.
- Most of the investigated anions showed a millimolar affinity for GsaCAβ, which is the typical inhibitory profile for this type of compounds. Indeed, K_I_-s in the range of 1.9–91 mM were measured for the following anions: fluoride, chloride, bromide, cyanate, thiocyanate, nitrite, bisulphite, bisulphide, tellurate, perosmate, divanadate, perrhenate, perruthenate, selenocyanate, imidosulfonate, and trithiocarbonate. Many of these anions are known for their propensity to complex with metal ions and they also act as inhibitors of other CAs, including hCA I and II (Table 4)^13–17^^,^^69–71^.
- Sub-millimolar inhibition of GsaCAβ was observed for cyanide, stannate, peroxydisulfate, and fluorosulfonate, which showed K_I_-s in the range of 0.77–0.92 mM.
- The most effective GsaCAβ inhibitors were sulfamide, sulphamic acid (presumably acting as sulfamate^72^) and *N,N*-diethyldithiocarbamate, which showed K_I_-s in the range of 6.2–81 µM. These inhibitors incorporate two well-known zinc-binding groups (ZBGs) present in many efficient CA inhibitors: the sulfamoyl moiety (present in sulfamide and sulphamic acid^72^, which has been demonstrated using crystallography to coordinate the zinc ion from the CA active site^72^. The same inhibition mechanism was thereafter observed for dithiocarbamates and their derivatives^73–75^, which incorporate the CS_2_^−^ ZBG. The fact that simple derivatives possessing no organic scaffolds (sulfamide and sulphamic acid) or a very small and compact scaffold (as *N,N*-diethyldithiocarbamate) do inhibit GsaCAβ quite efficiently, prompts us to hypothesise that it might be possible to develop more efficient and selective inhibitors for this enzyme, with potential use as antiparasitic agents.

### 3.3. Molecular model of GsaCAβ

We created a predicted structural model of GsaCAβ using AlphaFold^50^. The model is highly similar to crystallographic structures of other β-CAs. AlphaFold evaluates the per-residue confidence score (pLDDT, between 0 and 100) to be higher than 90 (“very high confidence”) for 65.1% of the residues and higher than 70 (“confident”) for an additional 26.2% of the residues. The N-terminal region 1–30 contains only residues with pLDDT <90. Counting from H31 (just before β-strand β1) to the end of the sequence, 75.3% of the residues are of very high confidence and 19.2% are confident. The only regions with low-confidence residues (50 < pLDDT < 70) are the N-terminus (8 of the first 10 residues) and the irregular helix at 67 to 78. All pLDDT values in our model can be found at https://bit.ly/3qIMIv6 (Supplemental data).

**Figure 6.**
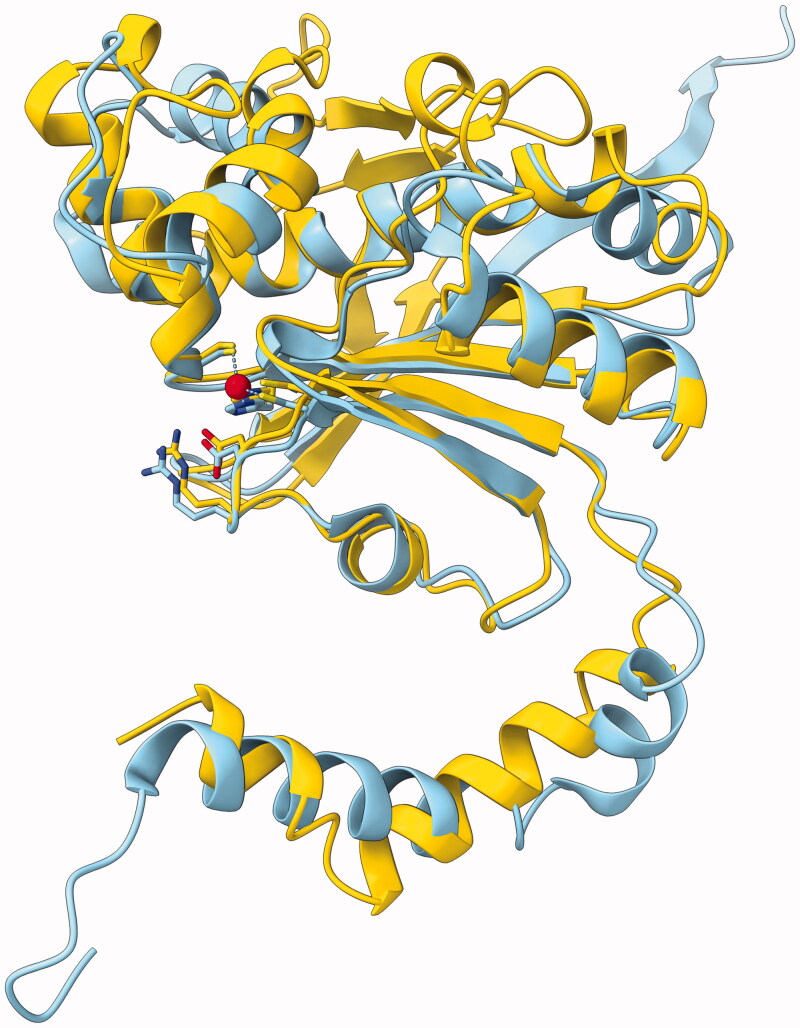
Molecular model of GsaCAβ. Our model constructed using AlphaFold, yellow, superimposed with pea β-CA (PDB 1EKJ, chain C), sky blue. The zinc ion of the catalytic site is shown in red.

In Figure 6 our model (yellow) is superimposed with pea β-CA (sky blue), with excellent fit over the core secondary structures and the loops of the catalytic site. The models of Figure 6 were superimposed with a strict iteration cut-off of 1.5 Å. There were 97 residue pairs left within the cut-off distance, with an RMSD of 0.858 Å, indicating excellent fit. Note that this RMSD is not a proper measure of the overall similarity of the two models, just between the subsets of the best-fitting residues. These structurally constant residues cover the parallel β-sheet formed of β-strands β2, β1, β3 and β7; α-helices α5 and α8 (on top of the β-sheet in Figure 6); the α-helix α6 extending after the HxxC motif; and the loops at the tips of β-strands β1 and β3 which form the catalytic site. Refer to Figure 3 for the numbering and locations of secondary structure elements.

The region 150–168 represents an insertion relative to the pea β-CA sequence. This region is seen near the top of Figure 6, predicted to form a β-hairpin-like structure containing β-strands β5 and β6. Despite the lack of similar loops in β-CA models in PDB, this region is mainly evaluated to be of high confidence.

To our knowledge, this is the second 3 D model for any platyhelmith β-CA to be made freely accessible, the first one being β-CA of *Schistosoma mansoni* in the AlphaFold database. Our model is available as a PDB file at https://bit.ly/3tPaNT2.

## 4. Conclusions

We report here the cloning and characterisation of a β-class CA identified in the genome of the Monogenean platyhelminth *Gyrodactylus salaris,* a parasite of Atlantic salmon and other economically relevant aquaculture fish species. Sequence analysis and successful modelling of the protein confirm its membership in the β-CA class. Sequence comparisons and the observed enzymatic activity also suggest that the N- and C-terminal sequences missing from our incomplete sequence are insignificant. This new enzyme, GsaCAβ, showed a low but significant catalytic activity for the physiological CO_2_ hydration reaction, with a k_cat_ of 1.1 × 10^5^ s^−1^ and a k_cat_/K_m_ of 7.58 × 10^6^ M^−1^ × s^−1^. This activity was inhibited by acetazolamide (K_I_ of 0.46 µM), a sulphonamide in clinical use, as well as by some inorganic anions and small molecules. Most investigated anions (fluoride, chloride, bromide, cyanate, thiocyanate, nitrite, bisulphite, bisulphide, tellurate, perosmate, divanadate, perrhenate, perruthenate, selenocyanate, imidosulfonate, and trithiocarbonate) were millimolar GsaCAβ inhibitors. Cyanide, stannate, peroxydisulfate, and fluorosulfonate, showed submillimolar range K_I_-s of 0.77–0.92 mM. Sulfamide (K_I_ of 81 µM), *N,N*-diethyldithiocarbamate (K_I_ of 67 µM) and sulphamic acid (K_I_ of 6.2 µM) were the most efficient GsaCAβ inhibitors. Correlated to the fact that there are very few non-toxic agents effective in combating this parasite, GsaCAβ is proposed as a new antiparasitic drug target for which effective inhibitors could be designed.

## Data Availability

Data files pertaining to this study, including the 3 D protein model, are available at https://github.com/MarttiT/G.-salaris-BCA. Individual supplementary files in this repository are indicated as bit.ly links in the text. Further data files and code are stored at https://github.com/thirtysix/Aspatwar.Gsalaris_BCA.
